# Supplementary material for: Activated Eosinophils Predict Longer Progression-Free Survival under Immune Checkpoint Inhibition in Melanoma
Source: Cancers (Basel). 2022 Nov 18;14(22):5676. doi: 10.3390/cancers14225676 (PMC9688620; doi:10.3390/cancers14225676)
Supplement: Supplementary file 1 [file cancers-14-05676-s001.zip › Supplementary Figure S10.pdf]

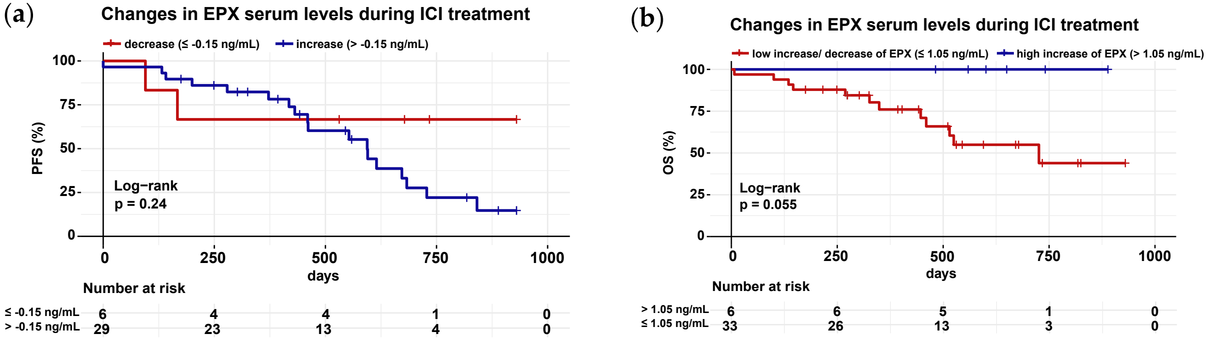
(a)
Changes in EPX serum levels during ICI treatment
decrease (≤ -0.15 ng/mL)
increase (> -0.15 ng/mL)
PFS (%)
100
75
50
25
0
Log−rank
p = 0.24
0
250
500
750
1000
days
Number at risk
≤ -0.15 ng/mL 6 4 4 1 0
> -0.15 ng/mL 29 23 13 4 0
(b)
Changes in EPX serum levels during ICI treatment
low increase/ decrease of EPX (≤ 1.05 ng/mL)
high increase of EPX (> 1.05 ng/mL)
OS (%)
100
75
50
25
0
Log−rank
p = 0.055
0
250
500
750
1000
days
Number at risk
> 1.05 ng/mL 6 6 5 1 0
≤ 1.05 ng/mL 33 26 13 3 0
