# Supplementary material for: Activated Eosinophils Predict Longer Progression-Free Survival under Immune Checkpoint Inhibition in Melanoma
Source: Cancers (Basel). 2022 Nov 18;14(22):5676. doi: 10.3390/cancers14225676 (PMC9688620; doi:10.3390/cancers14225676)
Supplement: Supplementary file 1 [file cancers-14-05676-s001.zip › Supplementary Figure S5.pdf]

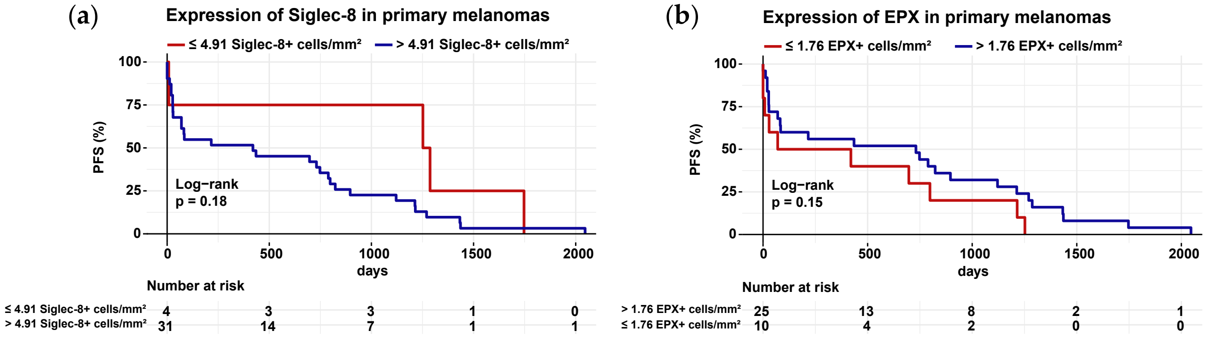

(a)
Expression of Siglec-8 in primary melanomas
≤ 4.91 Siglec-8+ cells/mm²
> 4.91 Siglec-8+ cells/mm²
PFS (%)
100
75
50
25
0
Log−rank
p = 0.18
0
500
1000
1500
2000
days
Number at risk
≤ 4.91 Siglec-8+ cells/mm² 4 3 3 1 0
> 4.91 Siglec-8+ cells/mm² 31 14 7 1 1
(b)
Expression of EPX in primary melanomas
≤ 1.76 EPX+ cells/mm²
> 1.76 EPX+ cells/mm²
PFS (%)
100
75
50
25
0
Log−rank
p = 0.15
0
500
1000
1500
2000
days
Number at risk
> 1.76 EPX+ cells/mm² 25 13 8 2 1
≤ 1.76 EPX+ cells/mm² 10 4 2 0 0
