# Supplementary material for: Activated Eosinophils Predict Longer Progression-Free Survival under Immune Checkpoint Inhibition in Melanoma
Source: Cancers (Basel). 2022 Nov 18;14(22):5676. doi: 10.3390/cancers14225676 (PMC9688620; doi:10.3390/cancers14225676)
Supplement: Supplementary file 1 [file cancers-14-05676-s001.zip › Supplementary Figure S7.pdf]

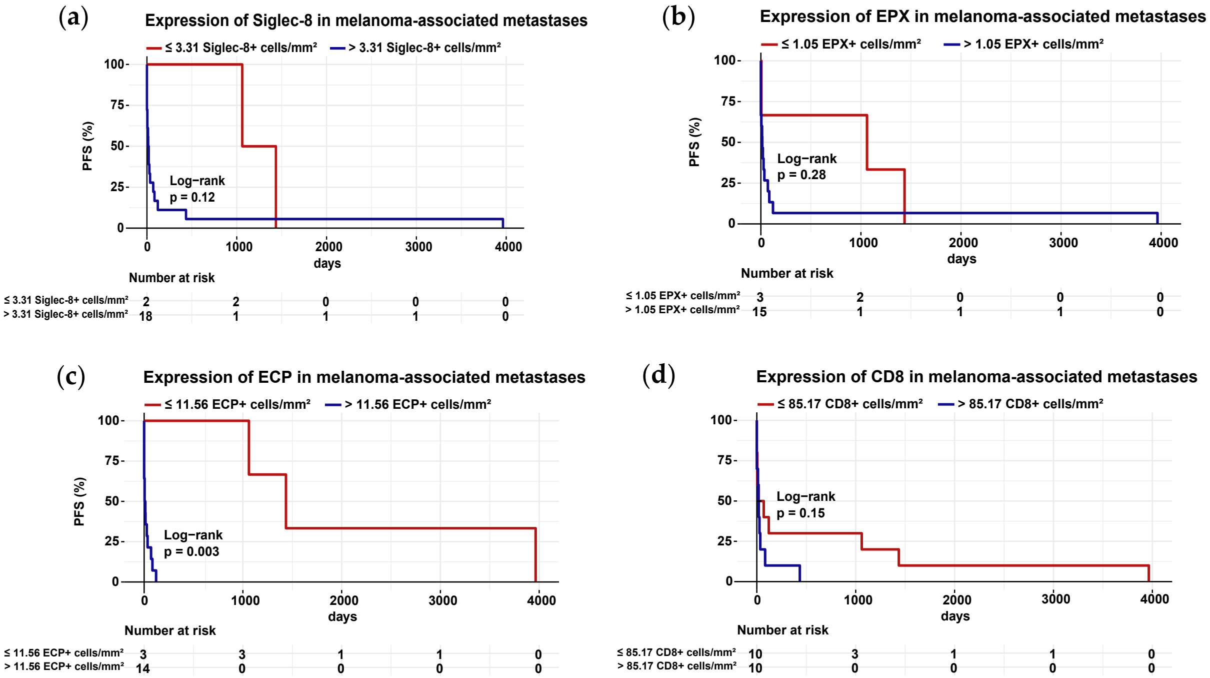

(a)
Expression of Siglec-8 in melanoma-associated metastases
≤ 3.31 Siglec-8+ cells/mm²
> 3.31 Siglec-8+ cells/mm²
PFS (%)
100
75
50
25
0
Log−rank
p = 0.12
0
1000
2000
3000
4000
days
Number at risk
≤ 3.31 Siglec-8+ cells/mm² 2 2 0 0 0
> 3.31 Siglec-8+ cells/mm² 18 1 1 1 0
(b)
Expression of EPX in melanoma-associated metastases
≤ 1.05 EPX+ cells/mm²
> 1.05 EPX+ cells/mm²
PFS (%)
Log−rank
p = 0.28
days
Number at risk
≤ 1.05 EPX+ cells/mm² 3 2 0 0 0
> 1.05 EPX+ cells/mm² 15 1 1 1 0
(c)
Expression of ECP in melanoma-associated metastases
≤ 11.56 ECP+ cells/mm²
> 11.56 ECP+ cells/mm²
PFS (%)
Log−rank
p = 0.003
days
Number at risk
≤ 11.56 ECP+ cells/mm² 3 3 1 1 0
> 11.56 ECP+ cells/mm² 14 0 0 0 0
(d)
Expression of CD8 in melanoma-associated metastases
≤ 85.17 CD8+ cells/mm²
> 85.17 CD8+ cells/mm²
Log−rank
p = 0.15
days
Number at risk
≤ 85.17 CD8+ cells/mm² 10 3 1 1 0
> 85.17 CD8+ cells/mm² 10 0 0 0 0
