# Supplementary figures and images for: Activated Eosinophils Predict Longer Progression-Free Survival under Immune Checkpoint Inhibition in Melanoma
Source: Cancers (Basel). 2022 Nov 18;14(22):5676. doi: 10.3390/cancers14225676 (PMC9688620; doi:10.3390/cancers14225676)

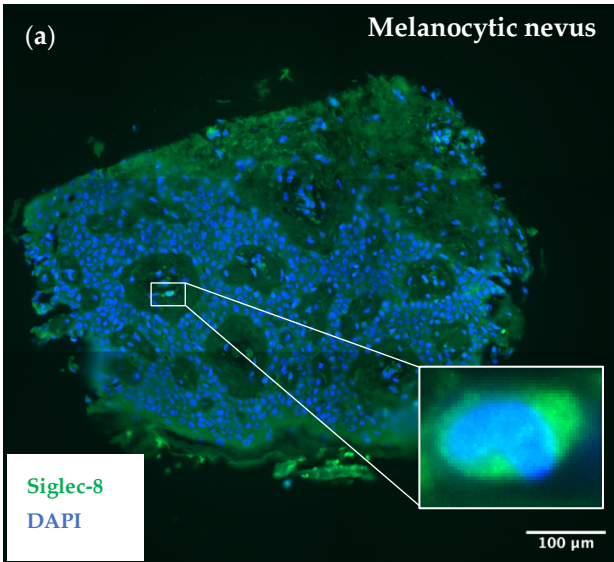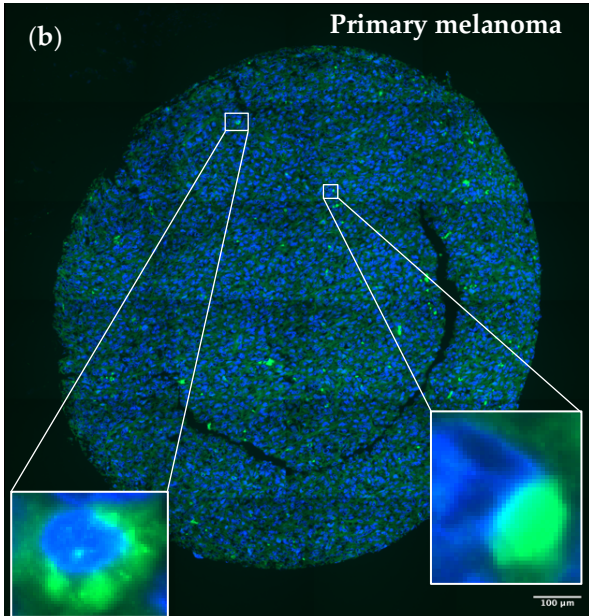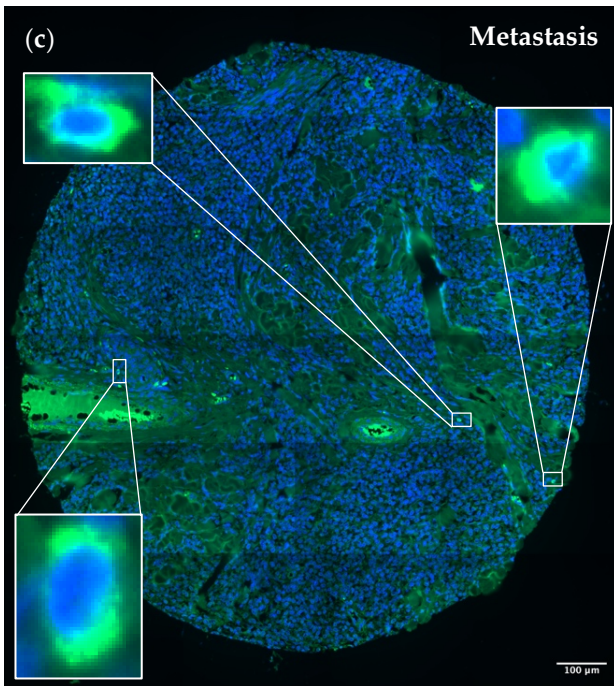

(d) Quantification of Siglec-8 expression

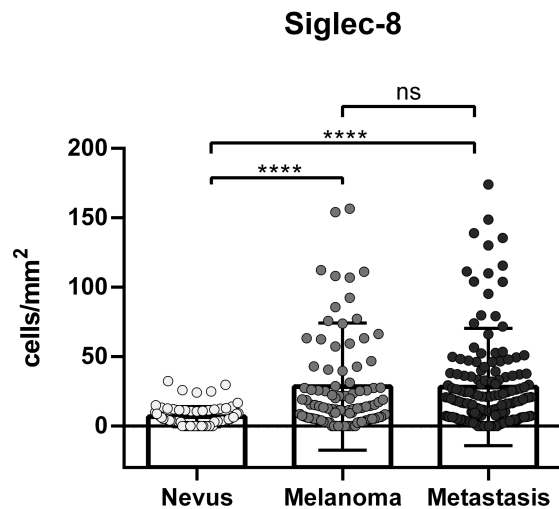

Supplement: Supplementary file 1 [file cancers-14-05676-s001.zip › Supplementary Figure S1.pdf]

(a) **Baseline peripheral-blood AEC**

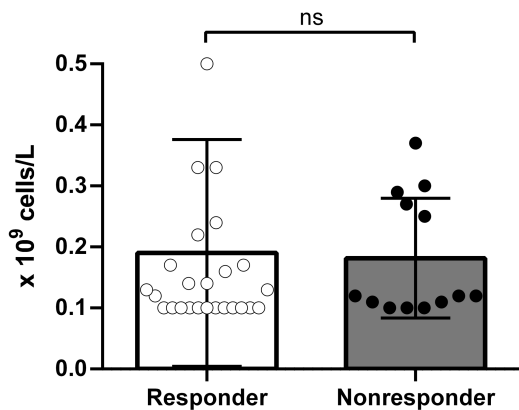

(b) **Baseline serum levels of ECP**

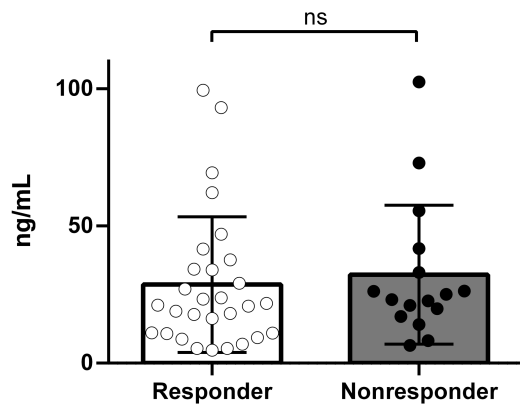

(c) **Baseline serum levels of EPX**

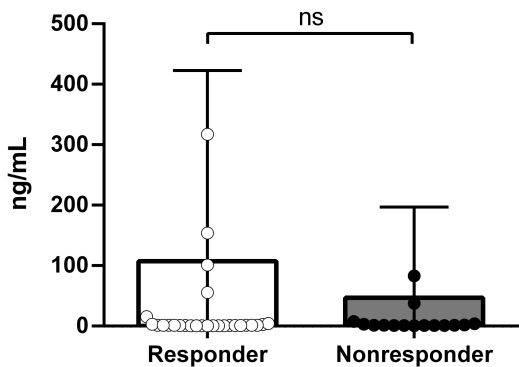

Supplement: Supplementary file 1 [file cancers-14-05676-s001.zip › Supplementary Figure S11.pdf]

(a) Peripheral-blood AEC during ICI

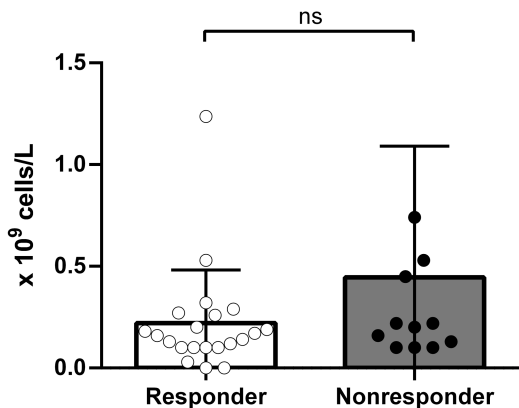

(b) ECP serum levels during ICI

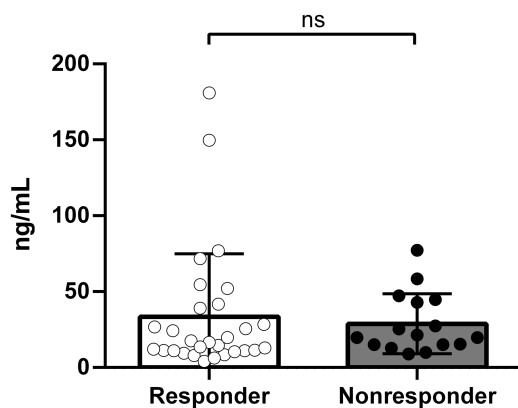

(c) EPX serum levels during ICI

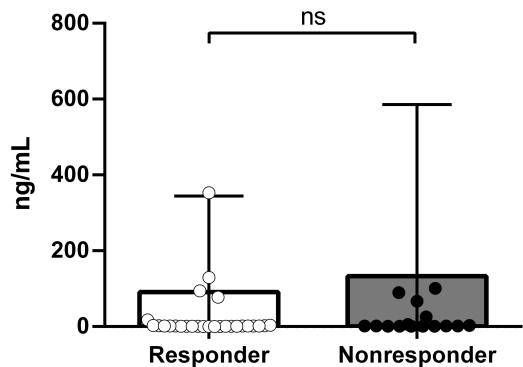

Supplement: Supplementary file 1 [file cancers-14-05676-s001.zip › Supplementary Figure S12.pdf]

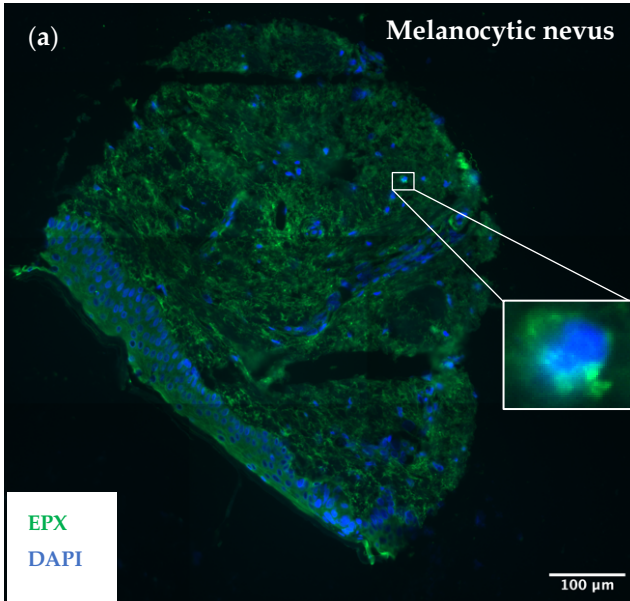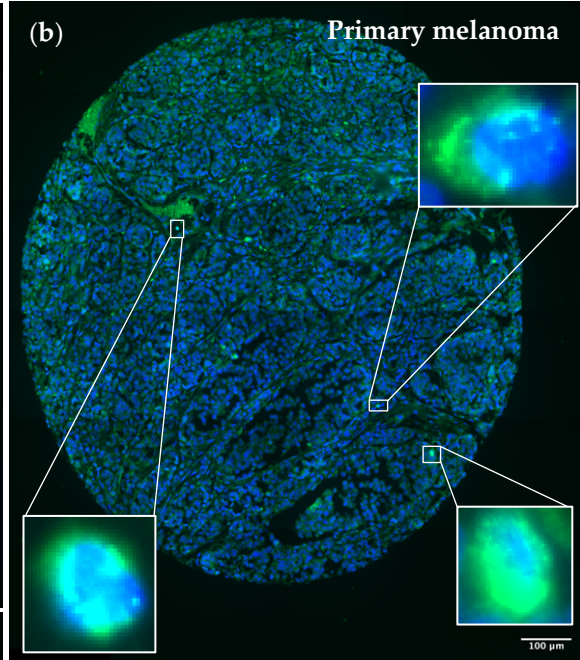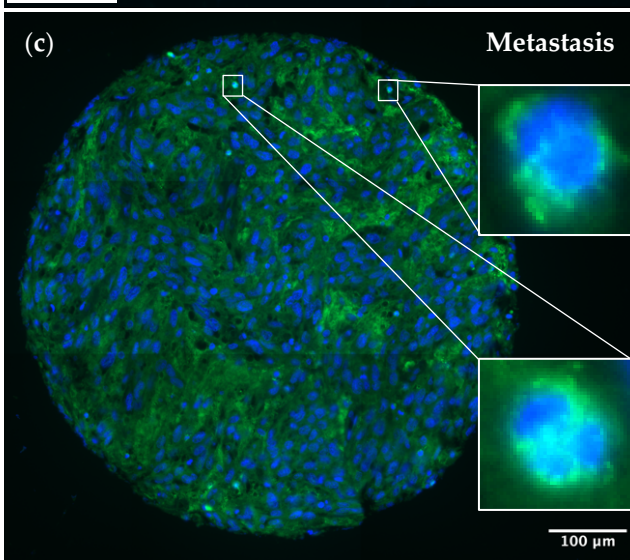

(d) Quantification of EPX expression

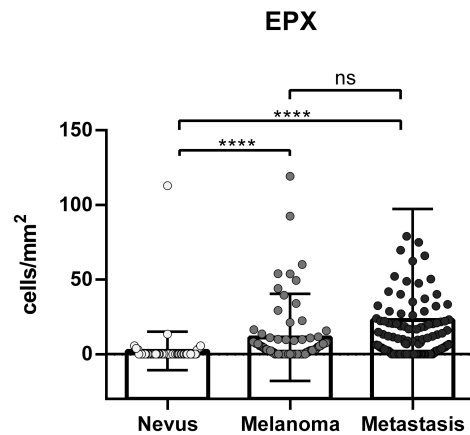

Supplement: Supplementary file 1 [file cancers-14-05676-s001.zip › Supplementary Figure S2.pdf]

# Primary melanoma

Siglec-8

CD8

DAPI

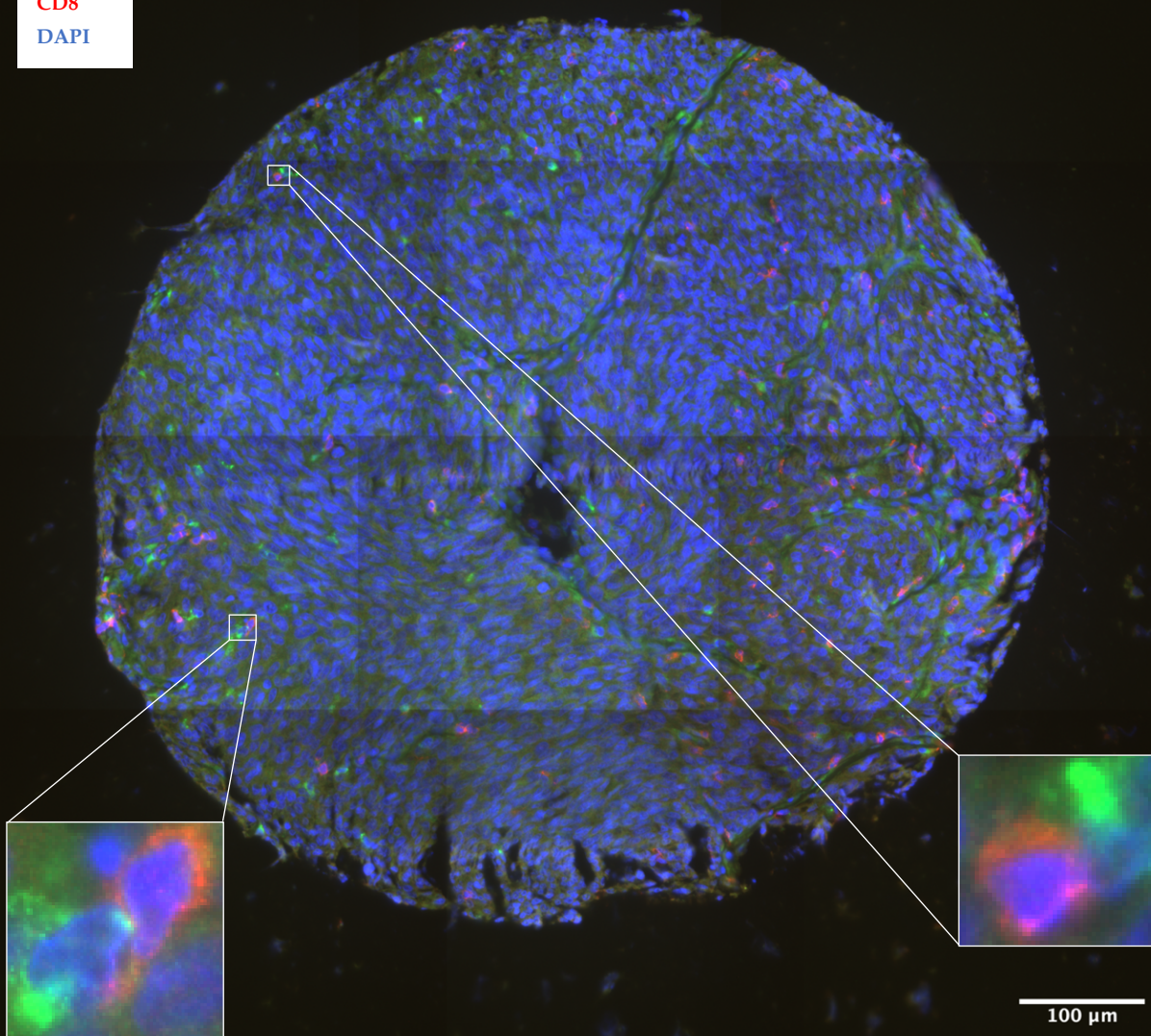

Supplement: Supplementary file 1 [file cancers-14-05676-s001.zip › Supplementary Figure S3.pdf]

# Metastasis

EPX  
CD8  
DAPI

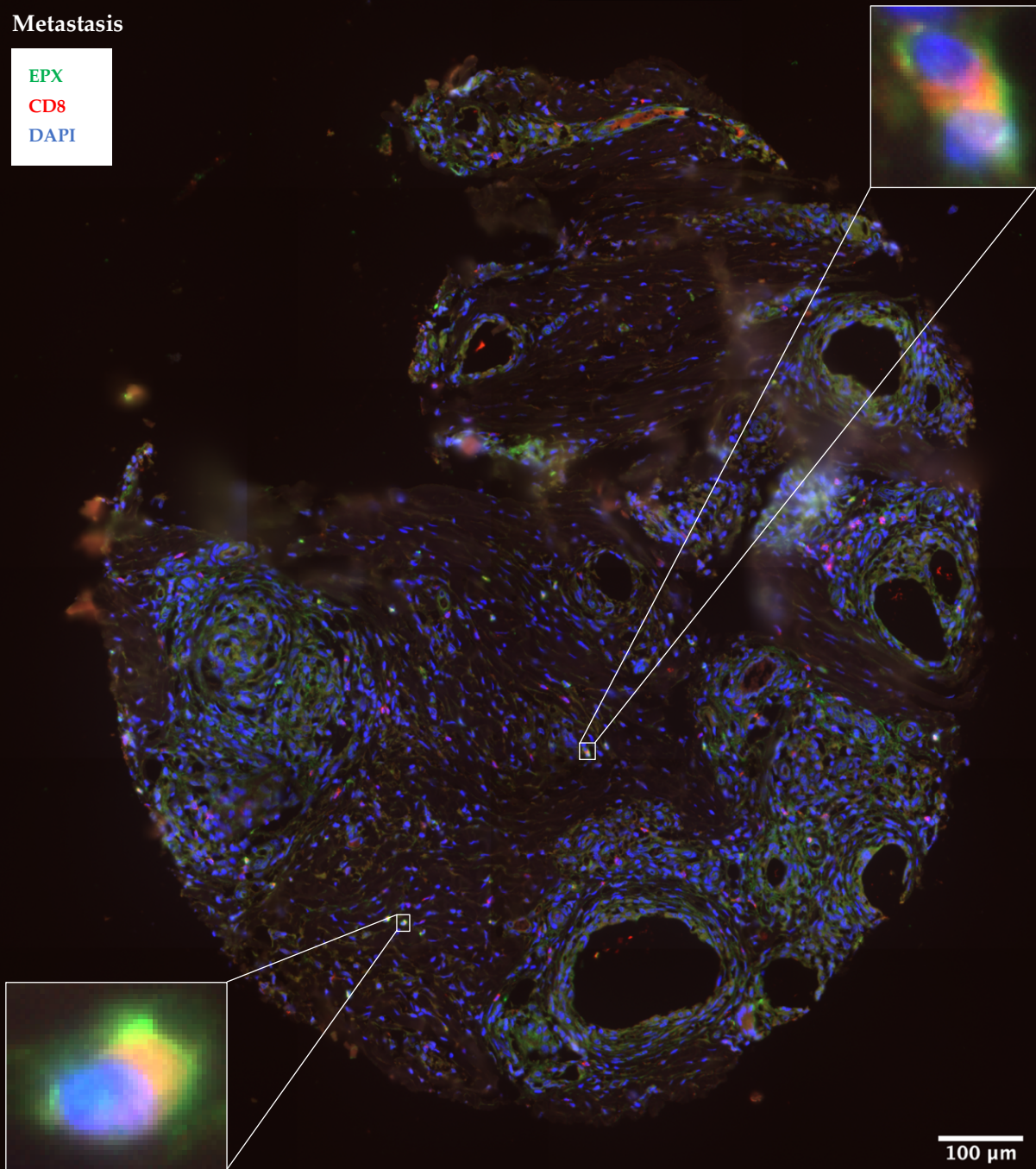

Supplement: Supplementary file 1 [file cancers-14-05676-s001.zip › Supplementary Figure S4.pdf]

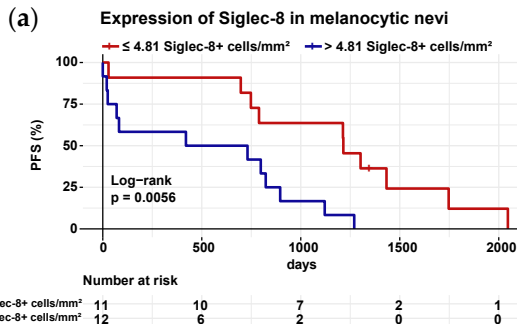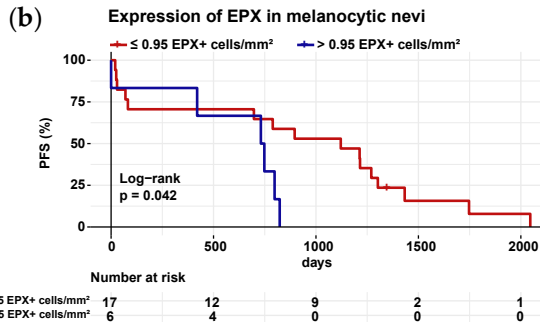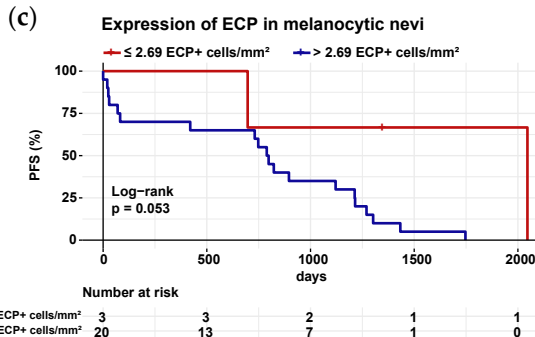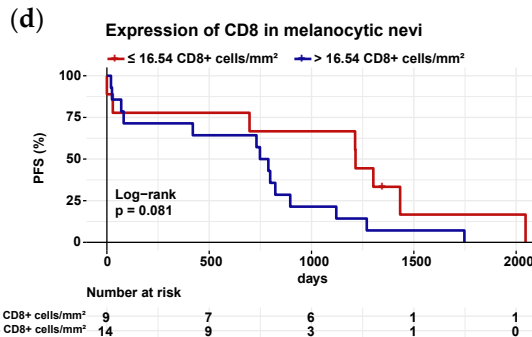

Supplement: Supplementary file 1 [file cancers-14-05676-s001.zip › Supplementary Figure S6.pdf]

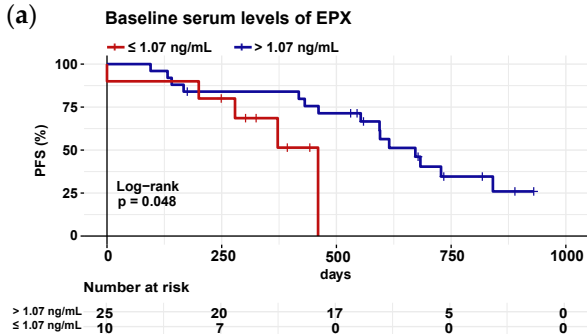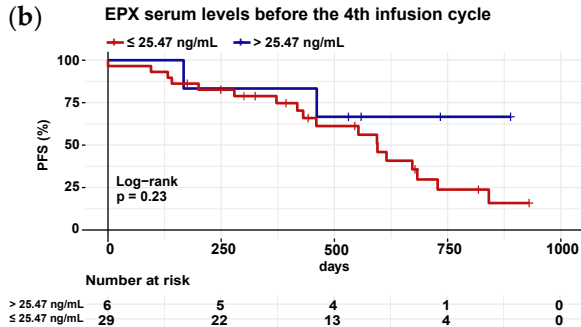

Supplement: Supplementary file 1 [file cancers-14-05676-s001.zip › Supplementary Figure S8.pdf]

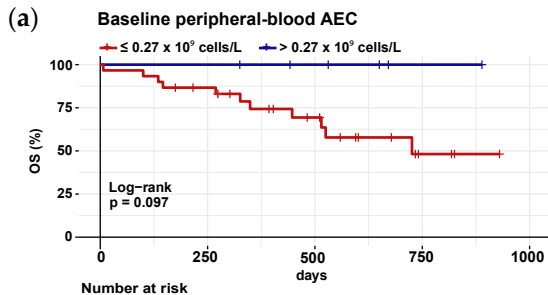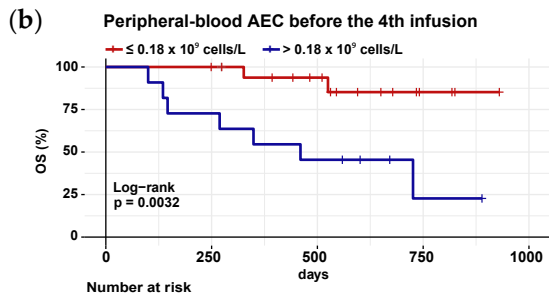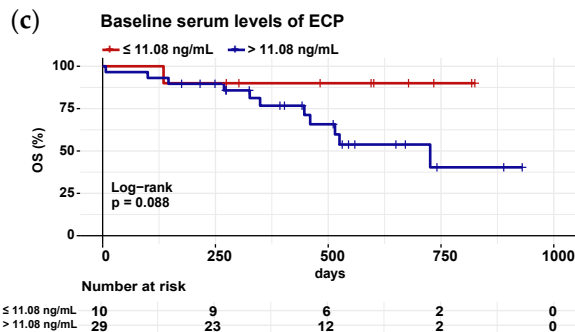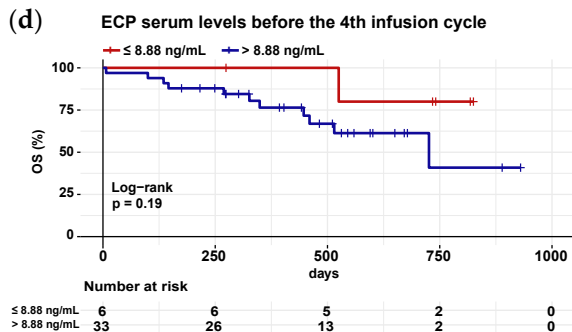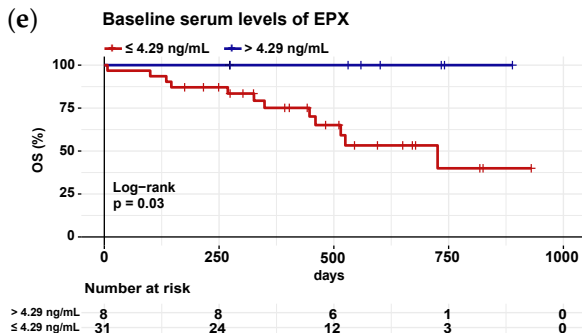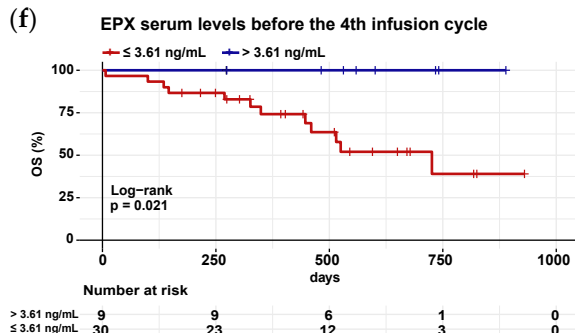

Supplement: Supplementary file 1 [file cancers-14-05676-s001.zip › Supplementary Figure S9.pdf]
